# Prevalence and associated factors of hypertension among adults with diabetes mellitus in northern Sudan: a cross-sectional study

**DOI:** 10.1186/s12872-021-01983-x

**Published:** 2021-04-10

**Authors:** Omer Abdelbagi, Imad R. Musa, Shaza M. Musa, Salim A. ALtigani, Ishag Adam

**Affiliations:** 1 Department of Pathology, Qunfudah Medical college, Umm-Al-Qura University, Al-Qunfudah, Kingdom of Saudi Arabia; 2 Department of Medicine, Royal Commission Hospital, Jubail Industrial City, Al Jubail, Kingdom of Saudi Arabia; 3 Faculty of Medicine, Najran University, Najran, Kingdom of Saudi Arabia; 4 College of Computer Science and Information Technology, Elsheikh Abdallah Elbadri University, Berber, Sudan; 5 Unaizah College of Medicine and Medical Sciences, Qassim University, Unaizah, Kingdom of Saudi Arabia

**Keywords:** Hypertension, Diabetes mellitus, Prevalence, Risk factors, Sudan

## Abstract

**Background:**

Hypertension and diabetes mellitus (DM), are highly prevalent worldwide health non-communicable diseases, and are associated with chronic complications. The co-existence of both conditions accelerates the related complications and increases morbidities and mortalities. A cross-sectional study was conducted in *Nahr an Nil* State (River Nile State) in Sudan between May and August 2018 to identify the prevalence of hypertension and risk factors among patients with DM in that region.

**Results:**

The median (interquartile) age of the 1,973 enrolled patients was 58.0 (50.0‒65.0) years, and 818 (45.6%) were males. The median (interquartile) duration of diabetes was 5.0 (3.0‒9.0) years. Of the 1,973 enrolled participants, 21.7%, 1.3%, 37.1%, and 39.9% were normal weight, underweight, overweight, and obese, respectively. Of 1,973 854 (47.6%) patients also had hypertension. Logistic regression analyses showed that elderly patients (adjusted odds ratio [AOR] = 1.03, 95%; confidence interval [CI] = 1.02‒1.04), males (AOR = 2.96, 95%; CI = 2.15‒4.07), employed patients (AOR = 1.92, 95%; CI = 1.38‒2.70), obese patients (AOR = 1.59, 95%; CI = 1.21‒2.08), and patients with diabetic foot (DF) (AOR = 2.45, 95%; CI = 1.72‒3.47) were at higher risk for hypertension. Conversely, patients with Type 2 DM (T2DM) (AOR = 0.63, 95%; CI = 0.50‒0.80) were at lower risk for hypertension. There was no significant association between overweight, uncontrolled DM, and hypertension.

**Conclusion:**

This study showed a high prevalence of hypertension among patients with DM. Notably, older age, male gender, employment, duration of DM, DF, underweight, and obesity were significant predictors of hypertension among patients with DM.

**Supplementary Information:**

The online version contains supplementary material available at 10.1186/s12872-021-01983-x.

## Introduction

Hypertension and diabetes mellitus (DM), which are both non-communicable diseases (NCDs), are global public health concerns [1]. NCDs are described as the invisible epidemic, with mortality that exceeds that of maternal, perinatal, and communicable diseases [2]. Hypertension globally and in Sub-Saharan Africa is expected to increase to 1.5 billion and 125.5 million, respectively, by 2025 [2]. Africa shows the higher prevalence of hypertension 33.3% in Northern Africa and (27%) in sub-Saharan Africa [3]. Likewise, the Middle East and North Africa have the highest world-age standardized diabetes prevalence (12.2%) [4]. Hypertension is a leading modifiable risk factor for premature death and disability worldwide [2]. The co-existence of hypertension and DM has been documented in previous studies [5–8]. The co-existence of hypertension and DM is associated a four-fold increase in mortality [9]. They are independent risk factors for micro-vascular diseases such as retinopathy, nephropathy and neuropathy [10]. Besides macrovascular complications that include coronary artery disease, myocardial infarction, stroke, congestive heart failure, and peripheral vascular disease [10]. Although, there is a difference in their pathophysiology, they show similar predisposing factors, such as heavy alcohol consumption, physical inactivity [11], obesity, genetic factors, and age ≥ 50 years [12]. Studies from Africa have shown a high prevalence of and complications related to hypertension in younger males (35–55 years), older females, the obese, and city dwellers with an unhealthy lifestyle [13, 14]. It has also been shown that unemployed status, male sex, aging, and nutritional transitions were determinants of hypertension in patients with DM [15]. High prevalence of hypertension (~ 25%) [16–18] and DM (20.8%) [19] has been reported in different parts of Sudan. Previous studies have shown different rates of hypertension among patients with DM in different African settings [10, 20, 21].

Few studies have addressed hypertension among patients with DM in Sudan [22]; hence, we conducted this study to assess the prevalence and associated factors of hypertension among adult patients with DM in northern Sudan.

## Materials and methods

### Study area

*Nahr an Nīl* (Nile River) State, one of 18 states in Sudan, is located in the north. It has seven localities, including Atbara, Berber, Ad Damar, Abu-Hamad, Shendi, El Matamah, and El Buhaira, an area of 122,123 km^2^ (47,152 mi^2^), and an estimated population of 1,511,442 citizens. The population includes Sudanese tribes who live beside the banks of the Nile River and practice agricultural and pastoral activities.

### Methods

A cross-sectional study was conducted in *Nahr an Nil* State in five localities. Patients with DM (T1DM and T2DM) which was defined according to diagnostic criteria of international diabetes federation; fasting plasma glucose > 7 mmol/l, two-hour plasma glucose > 11.1 mmol/l, HBA1C > 6.5% and random plasma glucose > 11.1 mmol/l in the presence of symptoms of hyperglycaemia [23]. After signing an informed consent form, all adult (aged ≥ 18 years) Sudanese residents of *Nahr an Nil* State (including both men and women) with DM were enrolled. Participants aged younger than 18 years, pregnant women, patients with poor cognitive function, and severely ill patients were excluded.

Data were gathered through a questionnaire that we developed for this study (Additional file 1). An OMRON 3 (with an appropriate size cuff) automated blood measuring device was used to measure blood pressure (BP), which was measured twice for each patient after resting for at least 10 min. The mean of the two (at an interval of 1–2 min) BP readings were calculated and recorded. If the difference between the two readings was > 5 mmHg, new measurements were taken until a stable reading was reached. The patient’s arm was maintained at the level of the heart.

Body mass index (BMI) was computed from the patient’s weight and height and categorized according to the World Health Organization classification as either underweight (< 18.5 kg/m^2^), normal weight (18.5–24.9 kg/m^2^), overweight (25.0–29.9 (kg/m^2^), or obese (≥ 30.0 kg/m^2^) [24].

The sample size of 1973 patients was calculated based on the previous prevalence (25%) of hypertension in Sudan [17, 18, 25]. Of the patients with DM who had hypertension (ratio of 1:4), 40% were obese. Additionally, 32.0% of the patients who did not have hypertension were obese. This sample (1973 patients) was calculated to detect a difference of 5% at *α* = 0.05 with a power of 80%. It was assumed that 10% of the patients might not respond or might have incomplete data. This study protocol followed STROBE guideline and its checklist is provided as (Additional file 2).

### Definition

Hypertension was defined as sustained high BP (systolic BP ≥ 140 or diastolic BP ≥ 90 mmHg) or reported regular use of antihypertensive medication [26]. Uncontrolled BP was defined as a systolic BP of ≥ 140 mmHg and/or a diastolic BP of ≥ 90 mmHg [27]. Controlled BP was defined as a systolic BP of < 140 mmHg and/or a diastolic BP of < 90 mmHg. A fasting blood glucose (FBG) level above 125 mg/dl was considered uncontrolled.

### Statistics

SPSS for Windows (version 22.0) was used to analyze the data. The chi-square test was used to compare the proportions between patients with DM who had hypertension and patients with DM who did not have hypertension. Continuous data were checked for normality using the Shapiro–Wilk test. A t-test and the Mann–Whitney test were used to compare the normally distributed and non-normally distributed data, respectively, between the two groups (hypertensive and non-hypertensive). Logistic regression analyses were performed by entering the dependent (hypertension) and independent variables (age, sex, location, BMI, duration of DM, and types of DM). The independent variables with a univariate *P* value < 0.20 were entered into the model. AORs and 95% CIs were calculated, with *P* < 0.05 considered statistically significant. Backward likelihood ratio adjustments were then used in the different models.

## Results

The median (interquartile) age of the 1973 enrolled patients was 58.0 (50.0‒65.0) years, and 818 (45.6%) were males. The median (interquartile) duration of diabetes was 5.0 (3.0‒9.0) years. The majority of the enrolled patients had their age ≥ 45 years. Of the  1973 enrolled participants, normal weight, underweight, overweight, and obese, were 21.7%, 1.3%, 37.1%, and 39.9% respectively (Table 1).

**Table 1:** Characteristic of diabetic patients in northern Sudan

Variables	Number	Percentage
*Age, years*		
< 35 year	119	6.6
35–44 years	159	8.9
≥ 45 years	1515	84.5
*Gender*		
Female	975	54.4
Male	818	45.6
*Location*		
Berber	405	22.6
Abu-hamad	303	16.9
Atbara	489	27.3
Ed Damar	329	18.3
Shendi	267	14.9
*Type of diabetes*		
Type 1	546	30.5
Type 2	1247	69.5
*Jobs*		
Non-employee	506	28.2
Employee	63	3.5
*Family history of hypertension*		
No	967	53.9
Yes	826	46.1
*Uncontrolled diabetes*		
No	416	23.2
Yes	1377	76.8
*Body mass index groups*		
Underweight	23	1.3
Normal	389	21.7
Overweight	666	37.1
Obese	715	39.9
*Diabetic foot*		
No	1559	86.9
Yes	234	13.1

Of the 1,973 enrolled DM patients, 854 (47.6%) also had hypertension. The median (interquartile) age was significantly higher in diabetic patients with hypertension compared with diabetic patients without hypertension (60.0 [51‒67.0] years vs. 57.0 [50‒64.0], respectively, *P* < 0.001]). There was no significant difference in the disease duration (5 years [4.0‒8.0] vs. 6 years [3.0‒10.0], respectively, [*P* = 0.552]) or in the location. Compared with patients without hypertension, a significantly higher number of hypertensive patients were in the age group of ≥ 45 years, male, employed, obese, had diabetic foot (DF), and had uncontrolled DM (Table 2).

**Table 2:** Comparing number (percentage) of characteristic of diabetic patients who had hypertension and who had no hypertension in northern Sudan

Variables	None- hypertensive (939)	Hypertensive (854)	*P* value
*Age*			
< 35 year	97 (10.3)	22 (2.6)	< 0.001
35–44 years	60 (6.4)	99 (11.6)	
≥ 45 years	782 (83.3)	733 (85.8%)	
*Gender*			
Female	573 (61.0)	402 (47.1)	< 0.001
Male	366 (39.0)	452 (52.9)	
*Location*			
Berber	199 (21.2)	206 (24.1)	0.009
Abu-hamad	159 (16.9)	144 (16.9)	
Atbara	242 (25.8)	247 (28.9)	
Ed Damar	173 (18.4)	156 (18.3)	
Shendi	166 (17.7)	101 (11.8)	
*Type of diabetes*			
Type 1	270 (28.8)	276 (32.3)	0.111
Type 2	669 (71.2)	578 (67.7)	
*Jobs*			
Non-employee	466 (49.6)	363 (42.5)	0.003
Employee	473 (50.4)	491 (57.5)	
*Family history of hypertension*			
No	513 (54.6)	454 (53.2)	0.538
Yes	426 (45.4)	400 (46.8)	
*Uncontrolled diabetes*			
No	238 (25.3)	178 (20.8)	0.025
Yes	701 (74.7)	676 (79.2)	
*Body mass index groups*			
Underweight	17 (1.8)	6 (0.7)	< 0.001
Normal	226 (24.1)	163 (19.1)	
Overweight	372 (39.6)	294 (34.4)	
Obese	324 (34.5)	391 (45.8)	
*Diabetic foot*			
No	879 (93.6)	680 (79.6)	< 0.001
Yes	60 (6.4)	174 (20.4)	

Logistic regression analyses showed that older patients (AOR = 1.03, 95%; CI = 1.02‒1.04), males (AOR = 2.96, 95%; CI = 2.15‒4.07), employed patients (AOR = 1.92, 95%; CI = 1.38‒2.70), obese patients (AOR = 1.59, 95%; CI = 1.21‒2.08), and patients with DF (AOR = 2.45, 95%; CI = 1.72‒3.47) were at higher risk for hypertension. By contrast, patients with Type 2 DM (T2DM) (AOR = 0.63, 95%; CI = 0.50‒0.80) were at lower risk for hypertension compared to type1 DM. There was no significant association between overweight, uncontrolled DM, and hypertension (Table 3).

**Table 3:** Factors associated with hypertension among diabetic patients in northern Sudan

Variable	Non-adjusted		Adjusted	
	OR (95%CI)	*P*	OR (95%CI)	*P*
*Location*				
Berber	1.95 (1.38‒2.74)	< 0.001	1.89 (1.35‒2.65)	< 0.001
Abu-hamad	1.71 (1.19‒2.46)	0.003	1.66 (1.16‒2.37)	0.005
Atbara	1.95 (1.40‒2.71)	< 0.001	1.89 (1.36‒2.61)	< 0.001
Ed Damar	1.75 (1.23‒2.49)	0.002	1.74 (1.22‒2.47)	0.002
Shendi	References		References	
Age, years*				
Median	1.03 (1.02‒1.04)	< 0.001	1.03 (1.02‒1.04)	< 0.001
Age, years (groups) *				
< 35 year	References		References	
35–44 years	6.96 (3.89‒12.45)	< 0.001	6.96 (3.89‒12.45)	< 0.001
≥ 45 years	3.63 (2.19‒6.03)	< 0.001	3.63 (2.19‒6.03)	< 0.001
*Duration of diabetes, years*				
Median	1.06 (1.03‒1.08)	< 0.001	1.06 (1.03‒1.08)	< 0.001
*Gender*				
Female	References		References	< 0.001
Male	2.88 (2.08‒3.98)	< 0.001	2.96 (2.15‒4.07)	
*Job*				
Non-employee	References		References	< 0.001
employee	1.88 (1.35‒2.63)	< 0.001	1.92 (1.38‒2.70)	
*Type of diabetes*				
Type1	References		References	< 0.001
Type2	0.65 (0.51‒0.82)	< 0.001	0.63 (0.50‒0.80)	
*Diabetic foot*				
No	References		References	< 0.001
Yes	2.46 (1.73‒3.49)	< 0.001	2.45 (1.72‒3.47)	
*Body mass index groups*				
Underweight	0.28 (0.10‒0.79	0.016	0.28 (0.10‒0.79)	0.016
Normal	References		References	
Overweight	1.10 (0.84‒1.45)	0.463	1.10 (0.84‒1.44)	0.476
Obese	1.57 (1.20‒2.06)	0.001	1.59 (1.21‒2.08)	0.001

*Were entered one by one

## Discussion

The prevalence of hypertension among patients with DM in this study was 45.6%. A similar prevalence (47.7%) of hypertension in patients with DM was reported in Khartoum, the capital of Sudan [22]. The prevalence of hypertension among patients with DM in our study (45.6%) was slightly lower than among DM patients in Nigeria (54.2%) [10] and Ethiopia (59.5%) [20], and it was much lower than among DM patients in Cameroon (86.2%) [21]. The association of hypertension and DM could be explained by hyperglycemia, insulin resistance, and dyslipidemia, which promote the atherosclerosis process [28]. This process can lead to narrowing of blood vessels and increased peripheral arterial resistance, which represent the hallmark of hypertension [29]. Moreover, insulin resistance could be associated with inappropriate activation of the renin–angiotensin–aldosterone system [30].

In this study, older patients with DM were at a 1.03 times higher risk for developing hypertension than younger patients. Several previous studies (e.g., in the capital of Sudan [22], Ethiopia [20], South Africa [15], and the Kingdom of Saudi Arabia [KSA]) [31] have reported that older patients with DM were at a higher risk for hypertension than patients without DM. Vascular remodeling, endothelial dysfunction, and vascular stiffness, which are common features in hypertension, are increased by aging [32]. Moreover, aging induces macrocirculatory changes that promote tissue hypoxia, reduce arteriolar and capillary density, and increase peripheral vascular resistance [33].

In the current study, employed patients had an increased risk for hypertension compared to non-employed participants, which is consistent with similar results from previous studies [34, 35]. Occupational noise, related to work environment was specifically found to increase the risk of developing hypertension [36]. Moreover, employment is associated with better educational achievement and greater access to healthcare [34]. Conversely, one study reported unemployed status as a potential risk for hypertension in South Africa [15].

The current study showed that males had a 2.96 times higher risk to have hypertension than females, which aligned with the results of previous studies [15, 31, 37]. Gender differences may be explained by sex hormones, sex-specific molecular mechanisms, and gender influences that affect both glucose and lipid metabolisms [38]. Moreover, the risk factors of carotid intima-media thickness, carotid plaque score, and pulse wave velocity differ in men and women and reflect useful atherosclerotic parameters and high BP [39].

Our study showed a significant association between hypertension and T2DM than those with T1DM. This was in accordance with the outcome obtained from previous studies [20, 40, 41]. The coexistence of T2DM and hypertension predicts poor control of hypertension [15]. Moreover, genetically instrumented T2DM was associated with the risk of hypertension [42].

The current study found obesity was a significant risk for developing hypertension than those with normal or overweight subjects, which supported similar findings of study conducted in Khartoum [22] and in the KSA [31]. Obesity is not only linked to the risk of hypertension, but it can also predict uncontrolled hypertension [43]. DM, hypertension, obesity (Additional file 2), and metabolic syndrome are modulQ3ated by genetics and epigenetics factors and influenced by lifestyle [44]. Moreover, metabolic syndrome is also associated with novel, significant, and additional pathways through which red blood cells participate in oxidative stress-dependent mechanisms to potentiate metabolic syndrome-associated vascular complications [45]. One recent study pointed to an inverse association of a mitochondrial DNA copy number with a higher risk for metabolic syndrome [46].

The limitations of this study include that some risk factors were not addressed, such as smoking, alcohol consumption, physical activity, diet behavior, and lipid profile and other chronic illnesses such as chronic kidney disease, sleep apnea and bronchial asthma.

## Conclusion

Our study documented a higher prevalence of hypertension among patients with DM in *Nahr an Nil* State in northern Sudan. The risk of developing hypertension was significantly associated with older age, male gender, employment, T2DM, duration of DM, presence of DF, and obesity.

## Supplementary Information

**Additional file 1.** Study Questionair.

**Additional file 2.** STROBE Checklist.

## Data Availability

The datasets used and/or analysed during the current study are available from the corresponding author on reasonable request.

## Abbreviations

AOR: Adjusted odds ratio
BMI: Body mass index
CI: Confidence interval
DF: Diabetic foot
DM: Diabetes mellitus
NCDs: Non-communicable diseases
BP: Blood pressure
LR: Likelihood ratio
SD: Standard deviation
WHO: World Health Organization
KSA: Kingdom of Saudi Arabia
