# Supplementary material for: Prevalence and associated factors of hypertension among adults with diabetes mellitus in northern Sudan: a cross-sectional study
Source: BMC Cardiovasc Disord. 2021 Apr 10;21:168. doi: 10.1186/s12872-021-01983-x (PMC8037914; doi:10.1186/s12872-021-01983-x)
Supplement: Supplementary file 1 — Additional file 1. Study Questionair. [file 12872_2021_1983_MOESM1_ESM.docx]

***Interview questionnaire for diabetic patient with with hypertension***

1. Age ……………………. Years

1. Sex

**□**Male **□** female

1. Locality

**□** Berber **□** Abu-Hamad **□** Atbara **□** Ed Damar **□**Shendi

1. Job

**□**Employee **□** non-employee

1. Type of diabetes

**□** Type I **□** Type II

1. Duration of diabetes ………………….years
2. Do you have family history of hypertension ?

**□**Yes **□** No

1. Have you ever taken regular anti-hypertensive drug or diagnosed as having blood pressure

**□** Yes **□**No

1. Presence of diabetic foot

**□** Yes **□**No

1. Height …………………………….. Cm
2. Weight ………………………………Kg
3. Blood pressure

| ………….mmgh |
| --- |

| ………mmgh |
| --- |

Systolic Diastolic
